# Immune microenvironment in autosomal dominant polycystic kidney disease

**DOI:** 10.1016/j.gendis.2025.101694

**Published:** 2025-05-26

**Authors:** Cheng Xue, Xinming Li, Chenchen Zhou, Changlin Mei, Zhiguo Mao

**Affiliations:** a Division of Nephrology, Shanghai Changzheng Hospital, Second Affiliated Hospital of Naval Medical University (Second Military Medical University), Shanghai 200003, China; b Outpatient Department, Yangpu Third Military Retreat, Shanghai 200433, China

**Keywords:** Autosomal dominant polycystic kidney disease, Complement, Immune cell, Inflammation, Macrophage

## Abstract

Autosomal dominant polycystic kidney disease (ADPKD) is a common hereditary renal disorder characterized by the progressive development of fluid-filled cysts within the kidneys, leading to renal dysfunction and potentially life-threatening complications. While ADPKD has long been considered a primarily genetic disorder, emerging evidence suggests that the immune microenvironment within the kidney plays a pivotal role in disease progression and severity. This review explored the intricate interplay between immune cells, inflammatory microenvironment, inflammatory pathways, complement system, and ADPKD, shedding light on the various immune components and mechanisms contributing to ADPKD pathogenesis. Key findings suggest that renal immune cell infiltration, inflammation, and the complement system could take part in cyst growth, renal fibrosis, and ADPKD progression. Inflammation, in particular, stands out as a prime candidate for therapeutic intervention. Moreover, recent studies have unveiled the involvement of immune checkpoints, such as PD-1 and its ligand PD-L1, in modulating the immune response within ADPKD kidneys. In conclusion, this review highlights the emerging paradigm shift in the understanding of ADPKD, emphasizing the pivotal role of the immune microenvironment in disease pathogenesis. Targeted therapies aimed at modulating immune responses and addressing immune-related checkpoints may hold promise for the development of novel treatments to improve the clinical outcomes of ADPKD patients.

## Introduction

Autosomal dominant polycystic kidney disease (ADPKD) is the most common monogenic hereditary kidney disease, characterized by the progressive enlargement of renal cysts, accounting for about 7%–10% of patients with end-stage kidney disease.^1^ ADPKD affects about 10 million individuals worldwide with an incidence of 1/2500–1/1000.^2^ The majority of ADPKD cases result from mutations in the polycystic kidney disease 1 (*PKD1*) or *PKD2* genes, which code for the proteins polycystin 1 (PC1) and polycystin 2 (PC2), respectively.^3^ Functional loss of the PC1/PC2 complex leads to abnormalities in a variety of intracellular signaling pathways, which contribute to cyst initiation and expansion. Cysts originate in tubules and are distinguished from simple tubule dilations by having increased numbers of cells expanding the wall beyond normal boundaries.^4^ One of the earliest consequences of PC1/PC2 dysfunction is impaired Ca^2+^ signaling due to defective mechanosensation in primary cilia.^5^ The resulting dysregulation of cAMP and calcium homeostasis drives epithelial cell proliferation, cyst fluid secretion, and mitochondrial dysfunction, ultimately leading to cellular stress and damage.^6^

ADPKD is characterized by the progressive formation and expansion of fluid-filled renal cysts, ultimately leading to chronic kidney disease and end-stage kidney disease in approximately 50% of affected individuals by the age of 60. While ADPKD is predominantly inherited due to mutations in either *PKD1* or *PKD2*, sporadic cases occur due to *de novo* mutations.^7^ Clinical presentation is highly variable, even among affected family members. Initial symptoms often include hypertension, flank pain, hematuria, and recurrent urinary tract infections, though some individuals remain asymptomatic for decades.^8^ Liver cysts, intracranial aneurysms, and cardiac valvular abnormalities are also common extrarenal manifestations. Children of affected individuals are often diagnosed earlier due to family screening with ultrasound or genetic testing, allowing for earlier implementation of risk-reducing measures such as blood pressure control, lifestyle modifications, and in some cases, early pharmacologic interventions. Familial cases, where a parent carries a *PKD1*/2 mutation, are often diagnosed earlier through routine family screening, allowing for earlier management with blood pressure control and disease-modifying therapies.^9^ In contrast, sporadic cases, arising from *de novo* mutations, are often diagnosed later, typically after symptoms like hypertension or renal dysfunction appear, which may limit early intervention opportunities and potentially lead to faster disease progression.

Given the progressive nature of ADPKD, recent research has focused on understanding how inflammation, immune responses, and the renal microenvironment contribute to cyst growth and fibrosis. Notably, while the immune microenvironment plays a well-recognized role in tumor development, its role in ADPKD, which shares similarities with tumors, has been largely overlooked. Inflammation is consistently observed in the kidneys of ADPKD patients and animal models, playing a dual role in regulating cyst growth and contributing to renal function decline.^10^, ^11^, ^12^, ^13^ However, despite these observations, the specific mechanisms by which the immune microenvironment influences ADPKD development and progression remain unclear. Understanding the precise roles and mechanisms of inflammation in ADPKD is crucial, and this review will explore the immune microenvironment in ADPKD pathogenesis, as well as potential therapeutic approaches.

## The immune system in ADPKD

The immune response in ADPKD involves the coordinated activity of both the innate and adaptive immune systems. The innate immune response, characterized as natural and non-specific, acts as the body's first line of defense against pathogens or altered endogenous molecules. It can also activate the adaptive immune system by signaling its initiation.^14^ The adaptive immune system, in contrast, involves specialized immune cells and processes that target specific pathogens, using mechanisms such as cytotoxic T cells and antibodies. Key players include T cells and B cells, which are activated by antigens presented by antigen-presenting cells and recognized through T-cell receptors or B-cell receptors.^15^

In ADPKD, a non-infectious environment, the immune system exhibits unique characteristics.^16^^,^^17^ A prominent feature of ADPKD is the accumulation of immune cells within the renal interstitium, which includes a diverse array of both innate and adaptive immune cells.^18^ Macrophages are involved in the early immune response, while T cells contribute more specialized functions in ADPKD.^18^ Understanding the roles of these cells is crucial for unraveling the immune landscape in ADPKD.

### Innate immune cells in ADPKD

#### Macrophages

**Figure 1:**
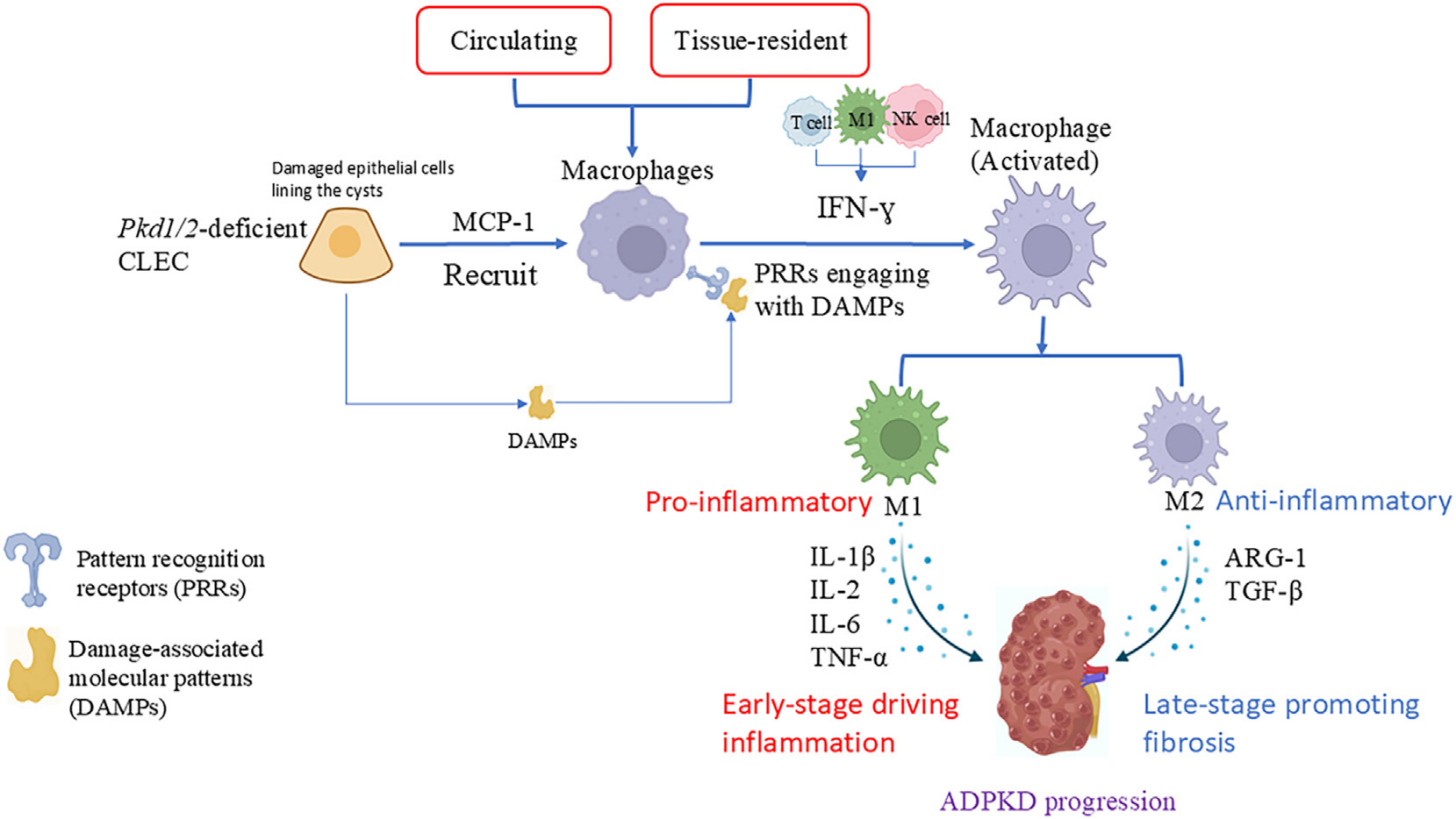
Macrophages in autosomal dominant polycystic kidney disease (ADPKD) pathogenesis. Macrophages in the kidney originate from circulating monocytes and resident macrophages. Chemokines, such as monocyte chemoattractant protein-1 (MCP-1), recruit macrophages to damaged tissues, leading to the polarization of macrophages into two phenotypes, M1 and M2. Macrophage infiltration contributes to the proliferation of cystic lining epithelial cells (CLECs) and the progression of polycystic kidney disease (PKD).

Kidney macrophages, originating from circulating monocytes and resident macrophages, form a complex network involved in ADPKD pathogenesis (Fig. 1). Tissue-resident macrophages, derived during development, are complemented by circulating monocytes in response to stimuli.^19^ Chemokines, notably monocyte chemoattractant protein-1 (MCP-1), recruit macrophages to damaged or infectious tissues, leading to the differentiation of monocytes into macrophages. These macrophages are key in kidney injury and repair and are among the most studied immune cells in ADPKD.^20^

Throughout life, the origins of kidney macrophages are diverse.^21^ In adult kidneys, renal-resident macrophages derive from i) fetal-generated macrophages, which express surface markers CD45^+^CD11b^low^F4/80^high^Ly6C^−^ and self-renew *in situ*,^22^ and ii) circulating macrophage progenitors, which express CD45^+^CD11b^high^F4/80^low^Ly6C^−^.^23^ In cystic congenital polycystic kidney (Cpk) mice, a well-established model of **autosomal recessive polycystic kidney disease**, characterized by early-onset cystic kidney disease, most macrophages expressed F4/80^+^Ly6C^−^.^24^ Fetal-generated macrophages persist into adulthood, though they are partially replaced by bone marrow-derived monocytes, with shifts occurring during development, adulthood, and disease.^25^

Macrophages are highly plastic and can polarize in response to local stimuli. *In vitro*, they are classified as M1 (pro-inflammatory) or M2 (anti-inflammatory and pro-fibrotic).^26^ In ADPKD, M1 and M2 macrophages play distinct roles in different stages of the disease.^27^ Macrophage infiltration contributed to the proliferation of the cystic lining epithelial cells (CLECs) and the progression of PKD in murine models.^28^ Depletion of macrophages using liposomal clodronate has been demonstrated to reduce cyst growth and improve renal function in *Pkd1*^fl/fl^:*Pkhd1*-Cre mice (a model **in which Pkd1 is specifically expressed in collecting ducts**).^28^ Additionally, studies have highlighted the abundance of M2-like macrophages in the kidneys of ADPKD patients and mouse models.^24^ CLECs from human ADPKD cysts could promote the differentiation of naive macrophages into a distinct M2-like phenotype *in vitro*.^24^ In our lab, two macrophage phenotypes were identified in rapid-onset ADPKD mice, and we confirmed that macrophage-CLEC interactions promoted cyst growth in *Pkd1*-deficient mice.^29^ Hypoxia within polycystic kidneys leads to L-lactic acid secretion from cysts, inducing M2 macrophage polarization. These M2 macrophages, characterized by high arginase 1 (ARG1) expression, promote cyst enlargement in *Pkd1*^*−*/−^ mice, suggesting ARG1 as a potential therapeutic target.

Renal interstitial macrophages, predominantly derived from circulating monocytes as the disease advances, suggest that chemokines and related factors promoting macrophage infiltration play a role in the progression of ADPKD. Genes involved in the innate immune response, including C–C motif chemokine ligand 5 (Ccl5), Ccl7, Arg1, and mannose receptor C-type 1 (Mrc1), were up-regulated in *Cpk* mice.^30^ Additionally, multiple macrophage chemotactic factors, such as migration inhibitory factor (MIF) and MCP-1, were overexpressed in cystic kidneys.^31^ Pkd1-deficient cells express high levels of macrophage chemoattractants, contributing to macrophage recruitment.^28^ Macrophage activation relies on signals from cytokines, ligands, and receptors. Interferon-gamma (IFN-γ) is the most potent macrophage activator, typically released in response to infections.^32^ Macrophages can be activated via pattern recognition receptors (PRRs) by interacting with pathogen-associated molecular patterns (PAMPs) or damage-associated molecular patterns (DAMPs).^33^ In the non-infectious microenvironment of the kidneys during the early stages of ADPKD, DAMPs serve as the primary drivers of macrophage activation. DAMPs, such as high mobility group box 1 (HMGB1) and S100 proteins, are associated with stress and are elevated in ADPKD patients.^34^^,^^35^ The initial release of DAMPs is closely linked to ciliary dysfunction-induced cellular stress. Loss of PC1/PC2-mediated Ca^2+^ signaling disrupts mitochondrial function and increases oxidative stress, leading to epithelial cell damage and death, which in turn triggers immune activation and chronic inflammation in cystic kidneys.^31^ Factors like reactive oxygen species and mitochondrial DNA also trigger macrophage activation in this context. Activated macrophages then play critical roles in inflammation, adaptive immune activation, and tissue remodeling in ADPKD.^36^ Additionally, DAMP-PRR-activated macrophages may exert their effects via inflammasomes, which release interleukin (IL)-1β and IL-18 upon caspase-1 activation.^37^

#### Roles of NK cells, NKT cells, and γδ T cells in ADPKD

**Figure 2:**
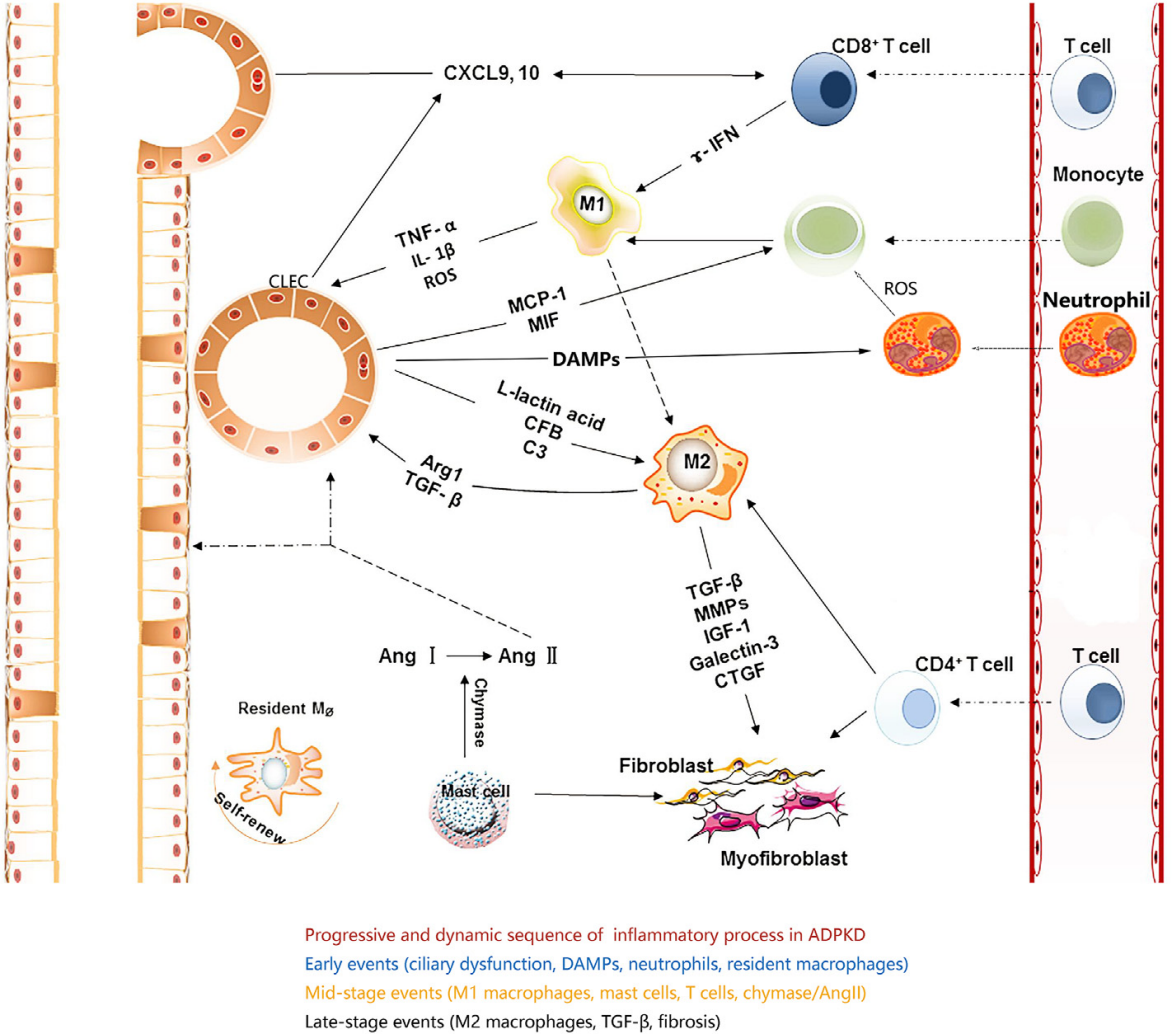
Immune cells in autosomal dominant polycystic kidney disease (ADPKD). Activated CD8^+^ T cells release interferon-gamma (IFN-γ), which promotes the polarization of macrophages to a proinflammatory phenotype (M1). Proinflammatory macrophages secrete cytokines, such as tumor necrosis factor-alpha (TNF-α), and promote the proliferation of cystic lining epithelial cells (CLECs). L-lactic acid is secreted from CLECs, which stimulates macrophages to present a profibrotic phenotype (M2). Furthermore, activated CD4^+^ T cells promote the polarization of macrophages to the M2 type. The M_2_-type macrophages with high expression levels of arginase 1 (ARG1) promote cyst enlargement. M2-type macrophages release profibrotic cytokines, such as transforming growth factor-beta (TGF-β), connective tissue growth factor (CTGF), platelet-derived growth factor (PDGF), and insulin-like growth factor (IGF), stimulate fibroblast and myofibroblast activation, and then promote fibrosis progression. Mast cells release chymase, which converts angiotensin I (Ang I) to Ang Ⅱ. Ang Ⅱ promotes renal tissue injury and fibrosis progression.

Natural killer (NK) cells, which constitute 5%–20% of circulating lymphocytes, eliminate stressed cells like tumors or virus-infected cells. NK cells, along with natural killer T (NKT) cells and γδ T cells, bridge innate and adaptive immunity.^38^ These cells can recognize glycolipids presented by CD1 family molecules, unlike conventional T cells that depend on major histocompatibility complex (MHC) molecules.^39^ In ADPKD, these cells could contribute to renal fibrosis.^40^ In ADPKD kidneys, stress signals may activate NK, NKT, and γδ T cells, triggering a response even in the absence of pathogens (Fig. 2). Glycolipids from proliferative or damaged cells could stimulate these cells, while NK cell receptors respond to stress-induced ligands like MHC class I-like molecules A and B (MICA/B), up-regulated under ADPKD conditions.^39^ Moreover, lipid mediators such as lysophosphatidic acid and sphingolipids may play a role in stimulating epithelial cell secretion, resulting in ADPKD progression.^41^ Lysophosphatidic acid has been linked to fibrosis and immune cell recruitment in kidney diseases, while sphingolipid signaling, particularly through sphingosine-1-phosphate (S1P), has been implicated in inflammation and cyst growth in PKD models.^42^ These lipid-derived signals could influence the activation and function of NK, NKT, and γδ T cells, further shaping the immune microenvironment in ADPKD. Although the roles of NKT and γδ T cells in ADPKD are not well-defined, they likely contribute to cyst growth and kidney injury through cytokine production, particularly IFN-γ, which could activate macrophages, establishing a cycle of inflammation and tissue damage.^43^

#### Mast cells in ADPKD

Mast cells are tissue-resident cells involved in responses against infection, wound healing, and inflammatory disease.^44^ The role of mast cells is well-studied in chronic renal inflammation, fibrosis lesions, and acute kidney injury progression.^45^ Mast cells mediated acute kidney injury progression through tumor necrosis factor (TNF).^46^ In 2003, the presence of mast cells and the potential for mast cell-initiated inflammatory processes were first reported in ADPKD cystic kidney.^47^ This study elucidated that mast cells within the inflammatory interstitium released chymase and provided an ACE-independent route of angiotensin II (Ang Ⅱ) generation in ADPKD. The high level of intrarenal ANG II may account for the interstitial macrophage infiltration in ADPKD.^47^

#### Neutrophils in ADPKD

Neutrophils are often the first responders to DAMP signals, and their role in ADPKD has been underexplored. Recent evidence suggested that neutrophilic infiltration occurs in polycystic kidneys, particularly under hypoxic conditions, where hypoxia-inducible factor 2 alpha (HIF-2α) stabilization has been observed in neutrophils.^48^ Additionally, systemic inflammatory markers such as neutrophil-to-lymphocyte ratio and mean platelet volume have been associated with ADPKD disease severity, suggesting a potential role for neutrophil-driven inflammation in disease progression.^49^

### Adaptive immune cells in ADPKD

The adaptive immune response is regulated by CD4/CD8 T cells and B cells, which coordinate cellular and humoral immunity, respectively. The principal distinction between innate and adaptive immunity lies in the specificity of antigen recognition facilitated by B-cell receptors or T-cell receptors. Conventional T cells (αβ T-cell receptors), collectively known as T cells, are diverse. CD4 T cells include subsets like T helper 1 (Th1), Th2, Th17, and regulatory T (Treg) cells. CD8 T cells function as cytotoxic T cells, killing target cells and releasing cytokines like IFN-γ.^50^ In ADPKD, where there is no infection, the adaptive immune response appears secondary (Fig. 2).

In 1989, lymphocyte was observed in slight infiltration in Han: SPRD rat kidney interstitial tissue.^51^ Lymphocyte infiltration is also observed in PKD mouse kidneys. However, those studies did not utilize lymphocyte-specific markers in any animal model. More than two decades ago, CD45, CD4, and CD8-positive lymphocytes were found infiltrating ADPKD kidneys.^52^ Recently, CD8^+^ T cells were found to play a protective role in C57BI/6 *Pkd1*^RC/RC^ mice (a hypomorphic model of slowly progressive ADPKD, mimicking milder human disease phenotypes), as depletion worsened ADPKD pathology.^53^ T cell recruiting chemokines C-X-C motif chemokine ligand 9/10 (CXCL9/10) and T cell-secreted cytokine IFN-γ increased in the kidney tissues of *Pkd1* mice.^53^ These findings suggest that CD8^+^ T cells may be a potential target for immunotherapy in ADPKD. In contrast, the roles of CD4^+^ T cells in ADPKD remain undefined. Ali et al ^54^ used T cell phenotyping in 72 ADPKD patients and revealed significantly raised CD3^+^ T cells, CD4^+^, CD8^+^, double-negative, and double-positive subsets, as well as significantly elevated IFN- and TNF-producing subsets of CD4^+^/CD8^+^ cells. Flow cytometry of kidney cells revealed that Pkd1^RC/RC^ animals had higher amounts of programmed cell death protein 1 (PD-1)/cytotoxic T lymphocyte-associated protein 4 (CTLA-4) on T cells and programmed cell death ligand 1 (PD-L1)/CD80 on macrophages and epithelial cells, which correlated with PKD severity.^55^ In ADPKD human cells and patient kidney tissue, PD-L1/CD80 was likewise increased compared with controls.^55^ Furthermore, Treg cell counts and suppressive markers CTLA-4, PD-1, and T cell immunoglobulin and ITIM domain (TIGIT) were considerably increased in the blood of ADPKD patients.^54^

The mechanisms driving the activation of T cells in ADPKD are largely unknown. It is suggested that the widespread cell proliferation in cystic kidneys, mediated by the DNA damage response, may play a role in the increased levels of T cells in ADPKD. Reports indicated that loss of PC1 impaired DNA damage response and induced cell proliferation in PC1-deficient kidney cells.^56^ PC1/2 expression in lymphocytes suggests that deficiency may increase DNA damage in ADPKD patients.^57^ Moreover, cytokines from innate immune cells may also influence adaptive immune activation. For example, inflammasome-activated macrophages release IL-1β, promoting T-cell activation.^58^

While various immune mechanisms are active in ADPKD, they do not occur simultaneously. Instead, the inflammatory response follows a progressive sequence. In the early stage, ciliary dysfunction and mitochondrial stress lead to DAMP release from injured epithelial cells, triggering neutrophil activation and recruitment of resident macrophages. As the disease advances to the mid-stage, M1 macrophages dominate the immune landscape, amplifying inflammation via TNF-α, IL-1β, and IFN-γ. Concurrently, mast cells release chymase, generating Ang II and promoting both fibrosis and further immune cell infiltration. In the late stage, a shift toward M2 macrophage polarization occurs, characterized by transforming growth factor-beta (TGF-β) and IL-10 secretion, contributing to interstitial fibrosis and functional decline.

## Inflammation in ADPKD

More than 30 years ago, Kenneth et al provided experimental evidence that environmental circumstances could modulate the expression of renal cystic disease in the *Pkd* rat model.^59^ They also reported that cytokines, such as TNF-α, IL-2, IL-1β, and prostaglandin E2, were found in cyst fluid from ADPKD patients and produced by CLECs.^60^ Meanwhile, another study found that cysts in the kidneys of *CFW*_*wd*_ mice, which were a mouse model of spontaneously occurring renal cysts, were provoked by the inflammatory environment.^61^ The deposition of IgG in cystic kidneys was demonstrated.^61^ Subsequent research using gene profiling of human polycystic kidneys has shown that genes associated with immune responses—such as complement 1s, IgG Fc receptor I, leukocyte common antigen, and CD2—were up-regulated in polycystic kidneys.^62^ A cross-species meta-analysis further supported the involvement of inflammatory processes in ADPKD pathogenesis.^63^ In PC1-deficient CLECs, overexpression of macrophage chemoattractants MCP-1 and CXCL16 has been observed. Supporting these *in vitro* findings, ADPKD patients' CLECs also overexpress multiple cytokines and complement system factors.^64^ While cyst formation and expansion are the central pathological features of ADPKD, interstitial inflammation is recognized as one of the earliest hallmark features of renal cysts.^16^ Clinical studies have verified early signs of inflammation in ADPKD, even when kidney function is still preserved, with inflammatory markers showing a graded relationship to kidney function levels.^65^ These findings were corroborated by observations that ADPKD patients exhibit significantly higher inflammatory indexes.^66^

### Inflammatory chemokines and cytokines in ADPKD

**Figure 3:**
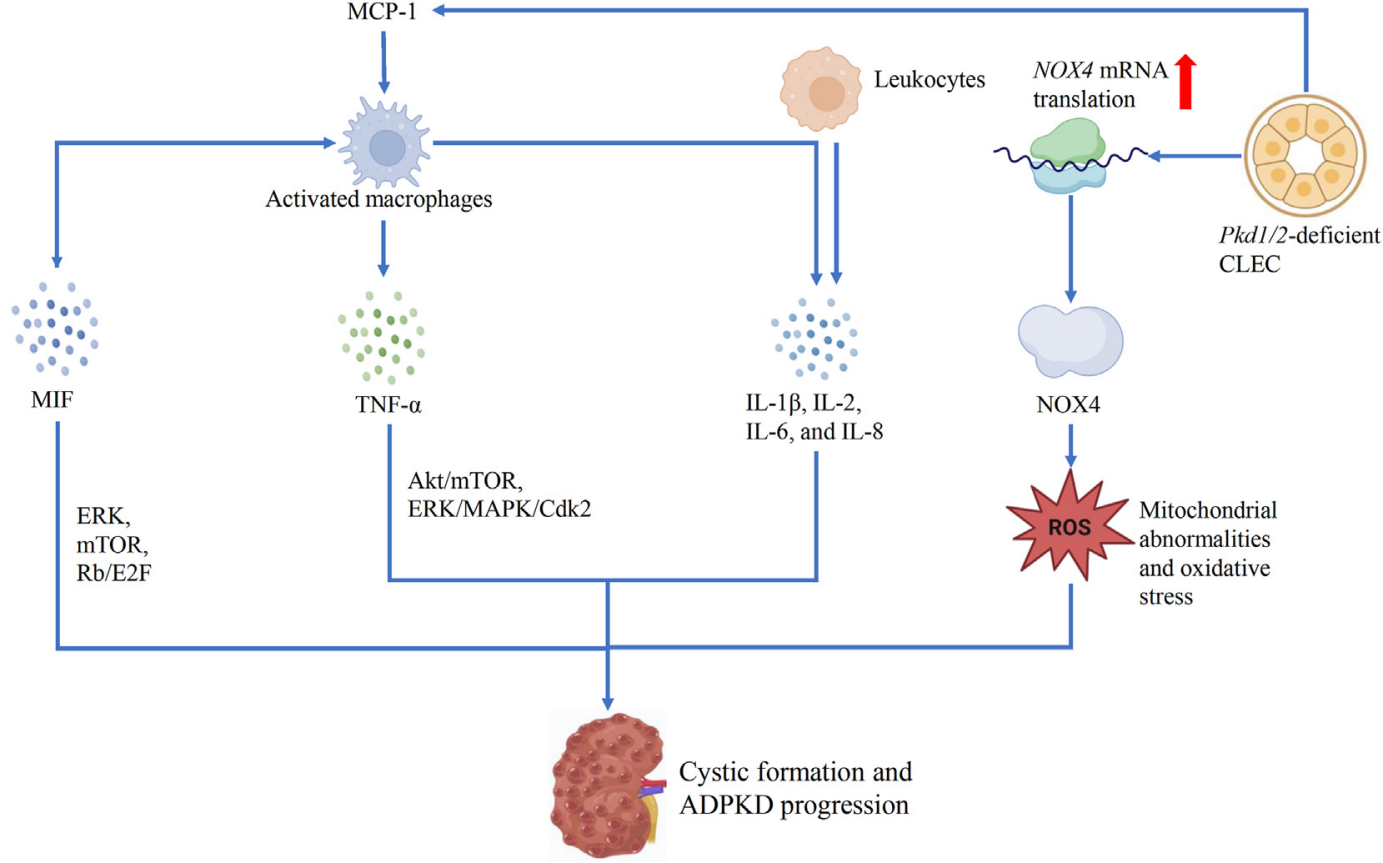
Inflammatory chemokines and cytokines in autosomal dominant polycystic kidney disease (ADPKD). Monocyte chemoattractant protein-1 (MCP-1), secreted by cystic lining epithelial cells (CLECs) and macrophages, mediates the recruitment of macrophages. Migration inhibitory factor (MIF) contributes to macrophage recruitment and the proliferation of CLECs by activating extracellular signal-regulated kinase (ERK), mammalian target of rapamycin (mTOR), and retinoblastoma protein (Rb)/E2F pathways. MIF also induced tumor necrosis factor-alpha (TNF-α) expression in renal epithelial cells, which created a positive feedback loop in ADPKD. TNF-α regulated CLEC proliferation through protein kinase B (Akt)/mTOR and ERK/mitogen-activated protein kinase (MAPK)/cyclin-dependent kinase 2 (Cdk2). Interleukins like IL-1β, IL-2, IL-6, and IL-8 also play crucial roles in cystic formation and disease progression. In early polycystic kidney disease (PKD), NAD(P)H-oxidase complex-4 (NOX4) expression was increased, which is linked to mitochondrial abnormalities and oxidative stress.

Chemokines are important in regulating immune cell behavior, including infiltration, activation, and polarization. Increased serum levels of cytokines and chemokines have been reported in experimental models of ADPKD and patients.^67^ Cytokines and chemokines could be secreted by immune cells and CLECs in ADPKD kidneys (Fig. 3).^68^

#### MCP-1

MCP-1 is a chemokine that particularly attracts monocytes and macrophages.^69^ MCP-1 mediates its inflammatory responses by binding to the receptor C–C motif chemokine receptor 2 (CCR2). MCP-1 is produced by diverse cell types, including macrophages, neutrophils, fibroblasts, endothelial cells, and epithelial cells.^68^ Increased renal expression of MCP-1 in ADPKD Han: SPRD rats (an PKD rat model, which develops cysts and progressive kidney dysfunction similar to human ADPKD) was associated with an increased number of interstitial macrophages.^70^ Urinary levels of MCP-1 were elevated in ADPKD patients and were associated with glomerular filtration rate decline and total kidney volume, suggesting its potential as a predictive tool in clinical practice.^71^ The ratio of urinary epidermal growth factor (uEGF)/MCP-1 was found to be a non-invasive predictor of the Mayo Clinic imaging classes of ADPKD.^72^ In the TEMPO 3:4 trial, tolvaptan administered to patients with ADPKD caused a sustained reduction in the urinary MCP-1.^73^

Bindarit, an oral inhibitor of MCP-1 synthesis, improved proteinuria and renal function but did not show effects on cyst progression in PKD rats.^74^ Excessive renal accumulation of monocytes/macrophages was lowered by bindarit by 41%.^74^ In another late-onset ADPKD mouse experiment, MCP-1 was up-regulated after *Pkd1* knockout, and pharmacologic inhibition of MCP-1 receptor CCR2 (INCB3344) slowed cyst growth.^75^ MCP-1 knockout decreased kidney MCP-1 level and macrophage number in *Pkd1* knockout mice.

#### MIF

Macrophage migration inhibitory factor (MIF) is a pleiotropic cytokine known for its critical role in recruiting both innate and adaptive immune responses.^76^ Recent studies have shed light on the functional roles and mechanisms of MIF in regulating CLEC proliferation and macrophage recruitment in ADPKD.^13^ In PC1-deficient murine kidneys, MIF was up-regulated in CLECs and accumulated in the cyst fluid of human ADPKD kidneys.^13^ MIF facilitated CLEC proliferation by activating extracellular signal-regulated kinase (ERK), mammalian target of rapamycin (mTOR), and retinoblastoma protein (Rb)/E2F pathways, boosting glucose uptake and ATP production, which in turn suppresses AMP-activated protein kinase signaling.^13^ This metabolic shift supports the increased energy demands of proliferating cells, driving cyst expansion. MIF also influences CLEC apoptosis through p53-dependent pathways. In PKD mice, MIF is essential for the recruitment and retention of renal macrophages, which further promote cyst growth and inflammation.^13^ Both genetic deletion and pharmacologic inhibition of MIF delayed cyst growth in various murine ADPKD models. MIF-driven macrophage recruitment correlated with increased MCP-1 and TNF-α levels.^13^ TNF-α induces MIF expression, which in turn amplifies TNF-α expression in CLECs, forming a positive feedback loop that intensifies cyst progression.^13^ Additionally, MIF binds to its receptor CD74, which modulates intracellular signaling and enhances MIF expression, further reinforcing this positive feedback loop in ADPKD.^77^ The interaction between MIF and CD74 not only promotes cyst growth but also exacerbates inflammation, contributing to disease progression.^77^

#### TNF-α

TNF-α is a key cytokine in systemic inflammation and promotes the recruitment of immune cells to injury or infection sites. TNF-α level was elevated in human ADPKD cyst fluids and increased significantly with age in *Cpk* mice.^78^ TNF-α in cyst fluid is secreted by activated macrophages and CLECs in ADPKD kidneys. Moreover, TNF-α contributed to cyst growth and enlargement during ADPKD progression.^10^ TNF-α signaling increased CLEC proliferation through protein kinase B (Akt)/mTOR and ERK/mitogen-activated protein kinase (MAPK)/cyclin-dependent kinase 2 (Cdk2) mediated inhibitor of DNA binding 2 (Id2) signaling.^12^ TNF-α can interfere with the localization of PC2 to both the plasma membrane and primary cilia by inducing the scaffold protein RAB11 family interacting protein 2 (FIP2), which in turn facilitates cyst formation in organ cultures and *Pkd2* mutant mice.^10^ Etanercept, an inhibitor of TNF-α, reduced cyst formation in *Pkd2*^+/−^ mice.^10^

#### Interleukins

The interplay of pro-inflammatory and anti-inflammatory mediators in ADPKD highlights a complex immune environment, where cytokines like IL-1β, IL-6, and IL-37 play key roles in cyst formation and inflammation regulation. High levels of IL-1β and IL-2 were detected in cystic fluids from polycystic kidneys.^60^ Moreover, the ADPKD patient group exhibited increased plasma concentrations of IL-6 and IL-8.^78^ Notably, urinary IL-18 stands as a well-established biomarker for acute and chronic kidney injury.^79^ While urinary IL-18 excretion remains mildly and consistently elevated in ADPKD, it does not show a correlation with changes in total kidney volume or kidney function.^80^ In a broader cohort encompassing subjects at various stages of ADPKD, the highest levels of plasma IL-6 and C-reactive protein were observed in the group with the most compromised kidney function.^65^ IL-1α and IL-1β, the two isoforms, were both elevated in ADPKD tissues.^81^ In the kidneys of *pkd1*^f/f^ KspCre + mice, we found activation of the IL-1 receptor suppressed TNF-α, mitigating controlled necrosis.^81^ Moreover, IL-1 receptor activation may exacerbate ADPKD by influencing controlled necrosis.^81^ Recent findings suggested that IL37, an anti-inflammatory cytokine, has the potential to reduce cyst burden and inflammation in ADPKD.^82^^,^^83^ This effect is achieved through modulation of the interferon signaling pathway in kidney resident macrophages.^82^

#### NOX4

The primary sources of endogenous reactive oxygen species in both renal tubular epithelial cells and endothelial cells are the nicotinamide adenine dinucleotide phosphate hydrogen (NAD(P)H)-oxidase complex-4 (NOX4) and the mitochondrial respiratory enzymes.^84^ In PCK rats, NOX4 expression was increased. Early PKD is linked to mitochondrial abnormalities and oxidative stress caused by NOX4, primarily in endothelial cells and CLECs.^84^ These findings suggest that NOX4 may serve as markers of mitochondrial injury and function, real-time biomarkers of oxidative stress for assessing disease severity, and treatment targets in ADPKD patients (NCT04630613).

### Activated complement system in ADPKD

The complement system is a part of the innate immune system.^85^ It can be brought into action by the adaptive immune system by generating antibodies. Three biochemical pathways activate the complement system: the classical complement pathway, the alternative complement pathway, and the lectin pathway. The alternative pathway might account for 80%–90% of total complement activation, even when initially triggered by the classical pathway or lectin pathway.^86^

**Figure 4:**
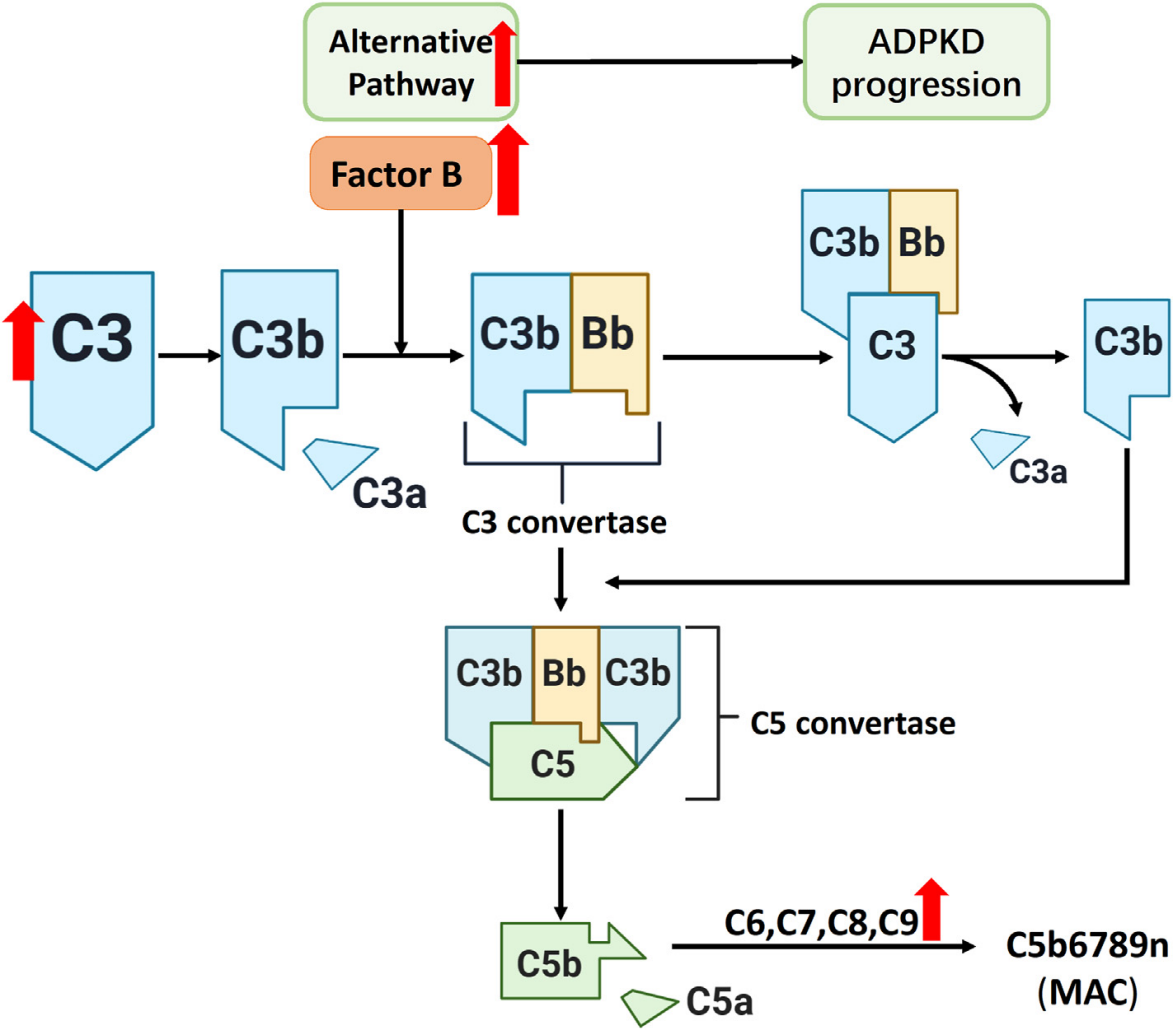
Activated complement system in autosomal dominant polycystic kidney disease (ADPKD). The complement system, especially the alternative complement pathway, plays a significant role in ADPKD pathogenesis. In ADPKD patients, complement component 3 (C3) is hyperactivated, and the levels of complement factor B (CFB) and C9 are elevated.

Growing evidence suggests that activation of the complement cascade may contribute to ADPKD (Fig. 4). The study by Mrug et al ^30^ has confirmed that innate immunity is involved in the progression of PKD mice, and particularly, abnormal complement component 3 (C3) activation is a key element. Burtey et al ^30^ also confirmed the overexpression of nine complement-component genes in the kidneys of Han: SPRD rats. The proteomic analysis of four samples of cyst fluid obtained postoperatively from excised kidneys in patients with end-stage kidney disease due to ADPKD found that 44 proteins included complement factors.^87^ The study of urine proteome in ADPKD patients also found a list of 155 proteins of different levels of the complement system factors and many others compared with healthy subjects.^88^ Mrug et al ^89^ further found that antigenic C3 was present in CLECs and that C3 activation fragments (iC3b) were present in renal cysts and urine from patients with ADPKD. A proteomic study of urinary extracellular vesicles revealed that complement-related proteins (C3 and C9) were more abundant in ADPKD.^90^

We found that excessive activation of the alternative complement pathway was associated with ADPKD progression.^91^ We screened the glycoproteome of urine samples from ADPKD patients and revealed that levels of complement factor B (CFB) and C9 increased with ADPKD progression. Immunostaining also showed that robust CFB signals were detected in CLECs from ADPKD patients.

To determine the role of complement in the disease progression, we evaluated the effect of the complement inhibitor rosmarinic acid in two separate rodent models of PKD, *Pkd1*^*−*/−^ mice and Han: SPRD Cy/+ rats. Compared with vehicle-treated *Pkd1*^*−*/−^ animals, rosmarinic acid-treated mice had significantly lower serum creatinine (50%) and blood urea nitrogen (78%) levels, two kidneys/body weight ratio (60%), and renal cystic index (60%). Similar results were found in Cy/+ rats. Lower numbers of Ki67-positive nuclei and inflammatory cells and reduced fibrosis were observed in both animal models upon treatment with rosmarinic acid.

We further explored the mechanism of CFB overexpression and alternative complement pathway activation in ADPKD.^92^ We observed that the overexpression of CFB in cystic kidneys was associated with increased Janus kinase 2 (JAK2)/signal transducer and activator of transcription 1 (STAT1) activity and enhanced expression of the polycystin-1 C-terminal tail (PC1-CTT). Moreover, STAT1 inhibition by fludarabine in CLECs suppressed ARG-1 expression induced by PC1-CTT, which suggested that PC1-CTT-induced macrophage activation into an M2 phenotype is mediated by STAT1 and CFB. In addition, our study showed that NF-kB is downstream of PC1-CTT and might partly mediate PC1-CTT-induced CFB expression.

Taken together, the above findings show that the complement activation, especially the alternative complement pathway is associated with ADPKD progression, and provides potential strategies that complement inhibitors may be useful agents to retard ADPKD progression.

### Pathways of inflammation in ADPKD

**Figure 5:**
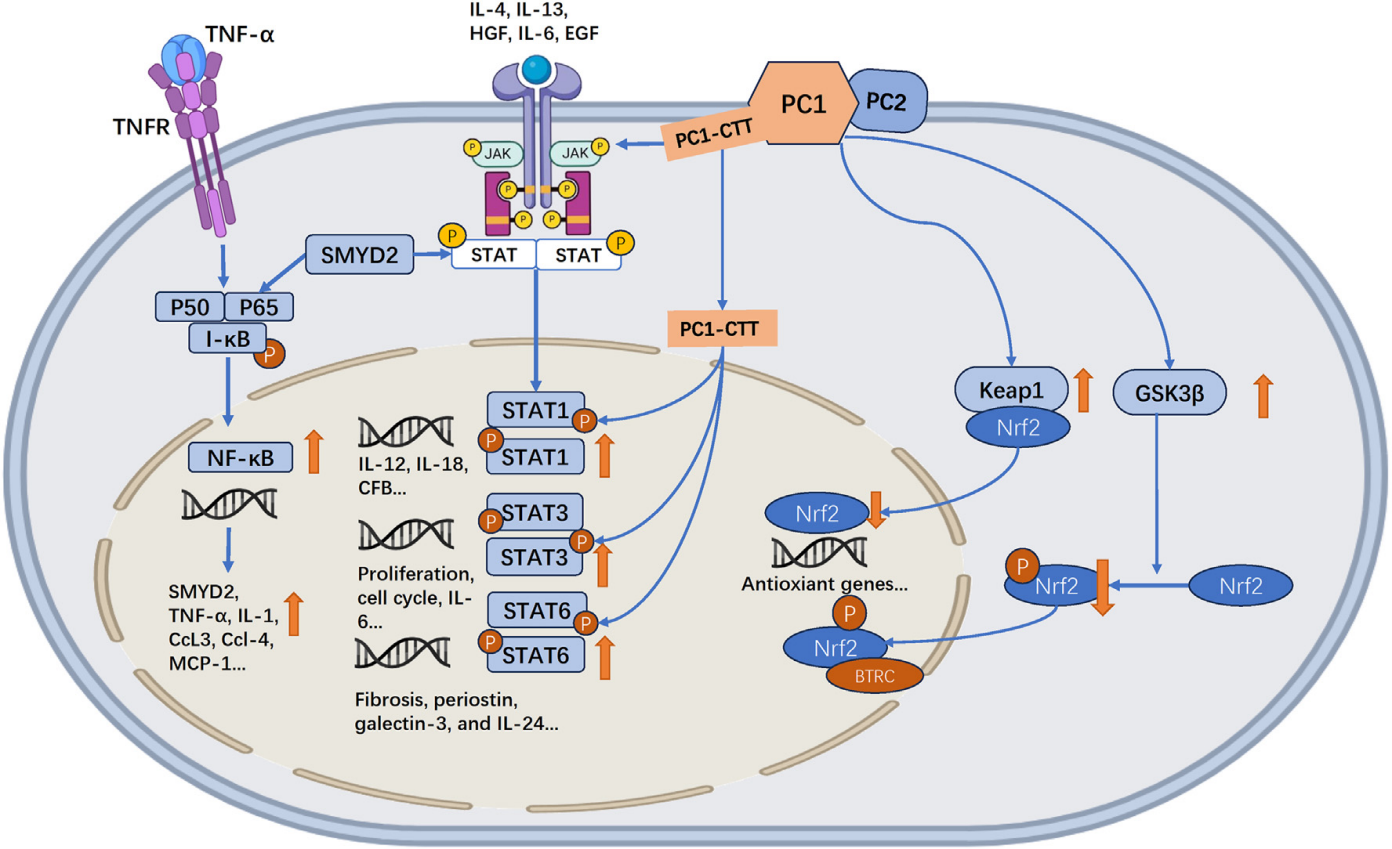
Pathways of inflammation in autosomal dominant polycystic kidney disease (ADPKD). In ADPKD, nuclear factor kappa B (NF-κB) is hyperactivated and is responsible for the transcription of pro-inflammatory cytokines and chemokines, such as tumor necrosis factor-alpha (TNF-α), interleukin-1 (IL-1), C–C motif chemokine ligand 2/3/4 (CCL2/3/4), monocyte chemoattractant protein-1 (MCP-1), and complement component 3 (C3). Polycystin 1 (PC1) activated a protein kinase C alpha (PKCα)-mediated NF-κB signal. SET and MYND domain-containing protein 2 (SMYD2) is responsible for the activation of signal transducer and activator of transcription 3 (STAT3) and the p65 subunit of NF-κB in ADPKD. Janus kinase (JAK)/STAT pathway plays a role in ADPKD pathogenesis by promoting cyst epithelial cell proliferation and immune responses. Polycystin-1 C-terminal tail (PC1-CTT) enhanced STAT1/3/6 activity. PC1-CTT regulated complement factor B (CFB) expression associated with JAK2/STAT1 activation. Kelch-like ECH-associated protein 1 (KEAP1) is the primary negative regulator of nuclear factor erythroid 2-related factor 2 (Nrf2). It binds to Nrf2 in the cytoplasm, facilitating its ubiquitination and subsequent degradation by the proteasome. Glycogen synthase kinase 3 beta (GSK3β) adds a second layer of control over Nrf2 by phosphorylating it. This phosphorylation promotes further ubiquitination and proteasomal degradation of Nrf2, similarly halting its signaling pathway.

The activation of nuclear factor kappa B (NF-κB) and STAT3 pathways, along with suppressed nuclear factor erythroid 2-related factor 2 (Nrf2) signaling and increased oxidative stress, up-regulates inflammatory cytokines and chemokines, driving cyst growth, inflammation, and fibrosis (Fig. 5). A gene profiling study of PKD1 cysts revealed elevated immune and inflammatory response genes, including those related to JAK-STAT and NF-κB pathways.^64^ Single-cell analysis identified failed repair of proximal tubular cells, proinflammatory fibroblasts, and collecting duct cells as key activators of proinflammatory signaling in ADPKD.^93^

#### NF-κB signaling

NF-κB signaling is activated and induces the inflammation response in ADPKD. NF-κB has long been recognized as a central regulator of inducible gene expression in the immune system, such as TNF-α, IL-1, CCL3, CCL4, and MCP-1.^94^, ^95^, ^96^ Furthermore, TNF-α can also activate the NF-κB signaling, and a feedback loop has been created.^97^ TNF-α induced its transcription through NF-κB and exerted a pro-survival effect on the cystic epithelium through NF-κB activation.^11^

NF-κB is hyper-activated and has high expression of phosphorylated p65 protein in PKD^−/−^ cells. NF-κB-dependent overexpression of Wnt signaling further promoted cystogenesis in PKD.^98^ Advanced glycation end product was highly expressed in CLECs and up-regulated intracellular NF-κB signaling in *Pkd2* transgenic mice.^99^ Manuela Banzi et al described a mechanism in which PC1 triggered the activation of NF-κB signaling through a protein kinase C alpha (PKCα)-mediated pathway.^100^ In human embryonic kidney cells, a PC1-CTT-dependent NF-κB activation was observed.

SET and MYND domain-containing protein 2 (SMYD2) carried out its function via methylation and activation of STAT3 and the p65 subunit of NF-κB in ADPKD, leading to increased CLEC proliferation.^101^ Two positive feedback loops that integrate renal inflammation in cyst development were established: SMYD2/IL-6/STAT3/SMYD2 and SMYD2/TNF-α/NF-κB/SMYD2. The SMYD2 inhibitor AZ505 was found to slow renal cyst growth in PKD mice.^101^ NF-κB also regulates complement gene expression in PKD.^102^

#### JAK/STAT signaling

JAK/STAT signaling is abnormally activated and plays a role in ADPKD pathogenesis by promoting CLEC proliferation, differentiation, transcription, and immune responses.^103^ Weimbs et al reviewed the regulation of STATs by PC1 and their role in PKD.^104^ The role of STAT1, 3, and 6 in PKD has been explored by several studies. Bhunia et al reported that *PKD1*/*2* regulated the activation of the JAK/STAT signaling pathway.^105^ JAK2 expression is elevated in PKD, and its inhibition suppresses cyst formation.^106^ PC1, in conjunction with PC2, induces JAK2 activation, leading to STAT1 and STAT3 activation, as shown in a full-length PC1 overexpression system. This study was conducted before the discovery of PC1-CTT.^107^^,^^108^ In ADPKD kidneys, PC1 tail fragments are overexpressed, including both 30 kDa (a full-length) and 15 kDa fragments (a half-length). The cleaved PC1 tail interacted with STAT6 and P100, enhancing STAT6 activity.^109^ PC1 regulated STAT activity by a dual mechanism,^110^ membrane-anchored PC1 activated STAT3, soluble PC1 tail co-activated STAT1, 3, and 6, and STAT3 activated required JAK2, which interacted with the PC1 tail. We found that the PC1-CTT-regulated CFB expression was associated with JAK2/STAT1 signaling activation.^92^ Upon activation by IL-4 and IL-13, STAT6 signaling plays a crucial role in driving M2 macrophage polarization, promoting myofibroblast transformation and accumulation, and contributing to fibrosis by regulating the production of extracellular matrix proteins.^111^ In PKD, IL-13 and STAT6 activity also mediate the expression and up-regulation of key profibrotic factors such as periostin, galectin-3 (Gal-3), and IL-24.^111^

#### Nrf2 signaling

Nrf2 is a key regulator of antioxidant and antiinflammatory pathways.^112^ Under normal circumstances, Kelch-like ECH-associated protein 1 (Keap1) binds to Nrf2 and aids its ubiquitination and proteasomal destruction while blocking its translocation to the nucleus.^113^ However, reactive oxygen species enhance the nuclear translocation of Nrf2, transcriptional upregulation of antioxidant enzymes, and transcriptional down-regulation of inflammatory cytokines while disrupting the connection between Keap1 and Nrf2. Glycogen synthase kinase 3 beta (GSK3β) phosphorylation, which similarly promotes ubiquitination and proteasomal destruction, provides a second way to stop Nrf2 signaling.^114^ In PKD, at least in its advanced phases, Nrf2 expression is low while Keap1 and GSK3β expression are both elevated.^114^ The severity of PKD is significantly exacerbated by Nrf2 deletion. In contrast, PKD is ameliorated when Nrf2 is activated, either by sulforaphane, which disrupts the Nrf2-Keap1 connection, or by a substance that inhibits GSK3.^114^ An ongoing observational study aimed to investigate the characterization of the Nrf2 response in ADPKD patients (NCT04344769).

### Inflammation in renal fibrosis of ADPKD

**Figure 6:**
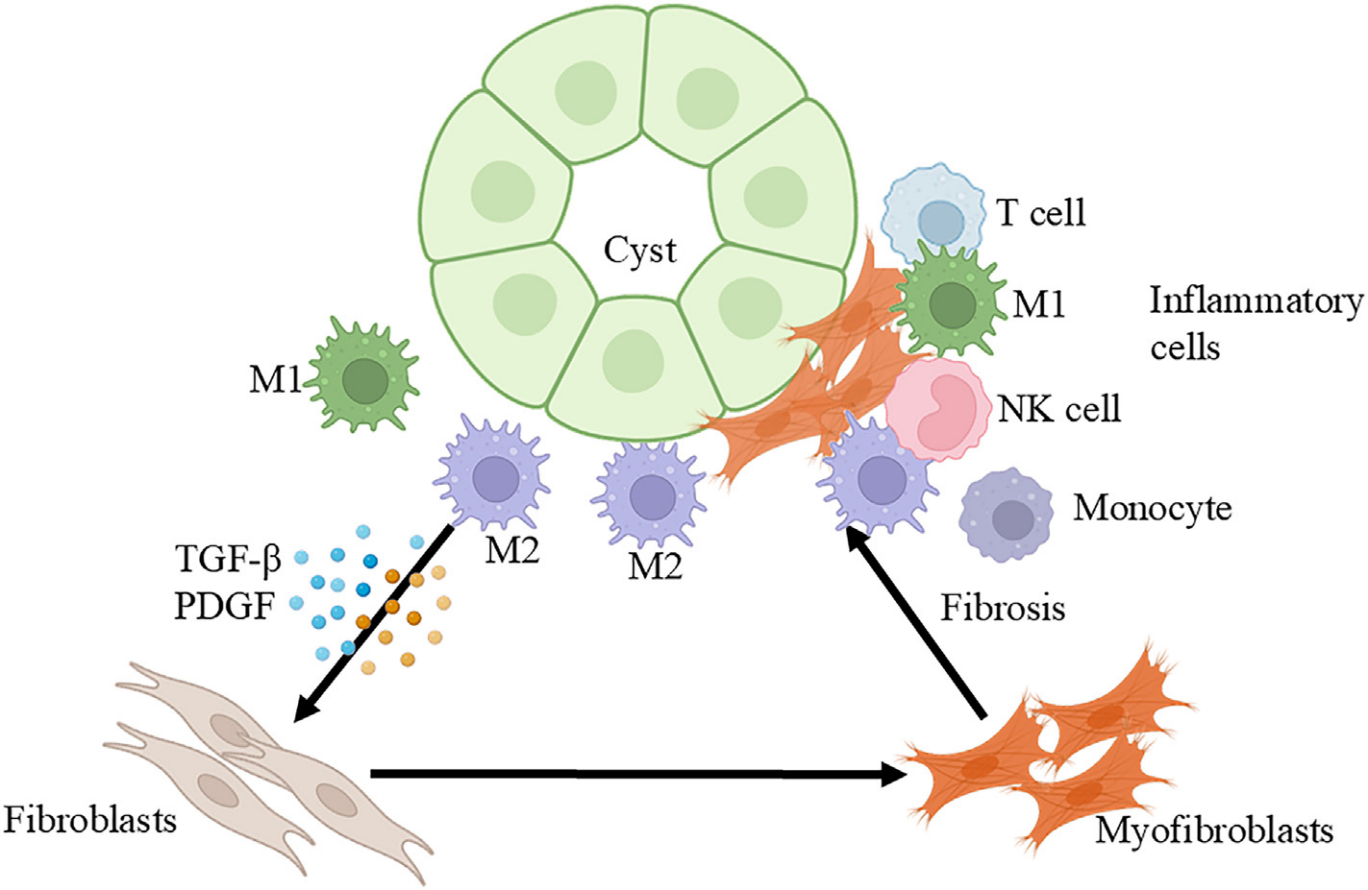
Inflammation in renal fibrosis of autosomal dominant polycystic kidney disease (ADPKD). In ADPKD, persistent inflammation leads to the recruitment of monocytes and the activation of macrophages. These activated macrophages then secrete and release profibrotic cytokines and growth factors such as transforming growth factor-beta (TGF-β) and platelet-derived growth factor (PDGF). These profibrotic factors stimulate the proliferation and survival of fibroblasts and myofibroblasts, contributing to fibrosis development and ADPKD progression.

ADPKD is linked to varying levels of interstitial fibrosis, a condition that represents the late stage of kidney disease (Fig. 6).^115^ Inflammation is regarded as a protective response in an attempt to eliminate the cause and promote kidney repair. However, ongoing inflammation, just considered as unresolved inflammation,^116^ promotes the formation of renal fibrosis. Kidney inflammation involves immune cells and activates intrinsic renal cells, followed by the production and release of profibrotic cytokines and growth factors that drive the fibrotic process.^117^ In ADPKD, interstitial inflammation is a chronic, persistent process, and this kind of unresolved renal inflammation is manifested as driving the recruitment of monocytes to the renal interstitium and promoting renal fibrosis continuously. Alternatively, activated macrophages promote renal fibrosis by producing profibrotic factors, Gal-3,^118^ insulin-like growth factor-1 (IGF-1),^119^ TGF-β1,^120^ and platelet-derived growth factor (PDGF),^121^ which promote myofibroblast proliferation and survival. Macrophages are a major source of matrix metalloproteinases (MMPs), including MMP-2, MMP-9, and MMP-12, which contribute to fibrosis development.^122^ MMPs can serve to both promote fibrosis and degrade extracellular matrix.^123^ There is a lack of research on the relationship between other immune cells and fibrosis in ADPKD.

## Potential and current immune therapies in ADPKD

Over the past decades, several studies have reported that experiments and trials targeted at inflammation could have a beneficial effect on delaying the progression of ADPKD (Table 1). Several immunotherapeutic compounds have been evaluated in animal models of PKD.^17^ Investigating immunotherapies aimed at correcting the dysregulated immune response in ADPKD, either by suppressing overactive cell types or enhancing protective ones, offers significant potential for improving patient outcomes.^124^ Therapies based on the order of disease occurrence, from early stage to later stage, are listed below.

**Table 1:** Summary of the functions and mechanisms of current immune treatments in autosomal dominant polycystic kidney disease (ADPKD).

Treatments	Year	Country	Source	Targets	Effects in ADPKD	Stages
Immune-checkpoint inhibitor^55^	2023	USA	Anti-PD-1 and anti-CTLA4 antibodies	PD-L1, CTLA-4	Enhances infiltration of activated CD8^+^ T cells and decreases CD4^+^ T cell frequency, resulting in reduced cyst growth in ADPKD	Animal experiment
Bardoxolone^132^	2019	USA	Semi-synthetic triterpenoid	Nrf2	Nrf2 activation; glomerular filtration rate protection	Ongoing phase 2 trial and phase 3 trial
FTY720^42^	2019	China	*Cordyceps Sinensis*	NF-κB, STAT	Inhibition of inflammation (IL-6, TNF-α)	Animal experiment
Resveratrol^131^	2016	China	Grapes, peanuts, berries, and their derivatives	NF-κB	Inhibition of inflammation	Animal experiment
MIF inhibitor^13^	2015	USA	Isoxazolines	Macrophages	Genetic deletion or pharmacological inhibition of MIF was shown to delay cyst growth	Animal experiment
Clodronate^24^	2013	USA	Clodronate liposome	Macrophages	Inducing macrophage exhaustion was associated with delayed cyst growth	Animal experiment
Etanercept^10^	2008	USA	2 p75 TNF receptors fused to the Fc portion of human IgG	TNF-α	Inhibition of TNF-α	Animal experiment
Triptolide^127^^,^^128^^,^^130^	2007; 2008; 2014	USA; China	*Tripterygium wilfordii Hook f*	NF-κB, STAT3	Induces cell apoptosis; regulates the cell cycle; inhibits TNF-α and IL-1β expression; reduces cyst growth and proteinuria	Ongoing phase 3 trial

Note: TNF-α, tumor necrosis factor-α; PC2, polycystin-2; NF-κB, nuclear factor kappa B; PD-1, programmed cell death protein 1; CTLA-4, cytotoxic T lymphocyte-associated protein 4; PD-L1, programmed cell death ligand 1; MIF, macrophage migration inhibitory factor; Nrf2, nuclear factor erythroid 2-related factor 2; STAT, signal transducer and activator of transcription; IL-6/1β, interluekin-6/1beta.

### Early-stage therapies

We utilized triptolide and resveratrol in ADPKD animal models and clinical trials. Triptolide, one of the active anti-inflammatory small molecules present in the plant extract, has been shown to inhibit TNF-α- and IL-1β-induced transcription at a step after NF-κB binding to DNA ^125^^,^^126^ while being a calcium-independent phenomenon. PC2 is regarded as a potential target for the biological activity of triptolide, and its therapeutic efficacy has been verified in a *Pkd1*^*−*/−^ mouse model.^127^ Treatment with triptolide significantly improved renal function at postnatal day 8 by inhibiting the early phases of cyst growth.^128^ Triptolide reduced cyst formation and cystic burden and preserved renal function in a neonatal to adult transition model.^129^ Our study revealed a long-term beneficial effect of triptolide in an adult rat model of PKD, probably through inhibition of the JAK2/STAT3 pathway. Our single-arm retrospective study found that triptolide could inhibit the development of cysts and reduce proteinuria in ADPKD patients with proteinuria.^130^ A phase 3 clinical trial in our department was performed (triptolide-containing formulation as treatment for autosomal dominant polycystic kidney disease; NCT02115659).

Resveratrol, a natural compound, reduced the levels of the MCP-1, TNF-α, and CFB in Cy/+ rat kidneys in parallel with the decreased activity of NF-κB (p50/p65).^131^ Our study confirmed that resveratrol inhibited cyst formation in the 3D cyst and zebrafish models.^131^ Resveratrol suppressed the expression of inflammatory factors and the NF-κB and mTOR pathways in both Cy/+ rats and ADPKD cells.^131^

### Mid-stage therapies

Neutralization of TNF-α presented an avenue to reduce inflammation overactivation in ADPKD. Etanercept, a biologic TNF-α inhibitor with FDA approval, is used to treat autoimmune diseases. Etanercept has been demonstrated to reduce the formation of renal cysts in *Pkd2*^*+/−*^ mice and serves as a decoy receptor for TNF-α.^10^ Bardoxolone, a Nrf2 activator, was found to raise estimated glomerular filtration rate during a 3-month follow-up according to a subanalysis of ADPKD patients in a clinical study of chronic kidney disease.^132^^,^^133^ There is now a phase III clinical study for bardoxolone in ADPKD (NCT03918447).

FTY720 (fingolimod) is a new immunomodulatory drug derived from *Cordyceps Sinensis*, a powerful sphingosine-1-phosphate receptor (S1PR) inhibitor.^134^ We discovered that FTY720 may prevent the activation of inflammatory pathways like STAT3 and NF-kB, decrease the production of pro-inflammatory cytokines like IL-6 and TNF-α, and inhibit the formation of renal cysts in PKD rats.^42^

Complement activation has been increasingly implicated as a driver of inflammation and interstitial fibrosis in ADPKD, particularly through the alternative complement pathway. Preclinical studies have shown that inhibition of CFB, either genetically or pharmacologically (*e.g.*, with rosmarinic acid), can reduce cyst growth, inflammation, and renal damage in ADPKD rodent models. These findings suggest that complement modulation may be a promising therapeutic strategy, especially during mid-stage disease when immune cell infiltration and fibrosis become more prominent. However, it is important to note that, to date, no complement-targeted therapies have advanced into clinical trials for ADPKD. Further studies are needed to evaluate their safety, efficacy, and potential translational value in human disease.

### Later-stage therapies

Inducing exhaustion of macrophages or genetically deleting MIF demonstrated delayed cyst growth and improved renal function, supporting the notion that targeting macrophages and related factors could be a viable ADPKD treatment strategy.^24^ Recently, immune checkpoint inhibitors have been demonstrated to reawaken CD8^+^ T cells and decrease tumor development in cancer. Similar to cancer, Emily et al have shown that CD8^+^ T cell depletion exacerbates PKD.^55^ In early-onset or adult-onset ADPKD animals, genetic PD-L1 deletion or therapy with an anti-PD-1 antibody did not affect PKD severity.^55^ However, inhibiting two immunological checkpoints with anti-PD-1 and anti-CTLA-4 improved PKD outcomes in adult-onset ADPKD mice, while neither monotherapy reduced PKD severity.^55^ Combination treatment enhanced kidney CD8^+^ T cell numbers/activation while decreasing kidney Treg numbers, which were proportional to PKD severity.^55^ Thus, immune checkpoint activation is a significant aspect of ADPKD and a possible new therapeutic target.

## Conclusion

The immune microenvironment plays a key role in ADPKD progression, involving a complex network of innate and adaptive immune cells, inflammatory cytokines, complement factors, and pathways. Both infiltrating and resident macrophages, along with other immune cells, could promote CLEC proliferation, creating a vicious cycle of inflammation and immune activity in PKD kidneys. Inflammation is central to this process, with pleiotropic effects influencing cyst growth, development, and renal fibrosis in ADPKD. Current knowledge of the immune microenvironment in ADPKD is limited. Targeted therapies that modulate inflammation, immune checkpoints, and complement factors may provide new treatment strategies for ADPKD in the future.

## CRediT authorship contribution statement

**Cheng Xue:** Writing – review & editing, Writing – original draft, Validation, Funding acquisition, Formal analysis, Data curation, Conceptualization. **Xinming Li:** Writing – review & editing, Writing – original draft, Validation, Investigation, Data curation, Conceptualization. **Chenchen Zhou:** Writing – original draft, Formal analysis, Data curation. **Changlin Mei:** Writing – review & editing, Validation, Funding acquisition. **Zhiguo Mao:** Writing – review & editing, Validation, Funding acquisition, Conceptualization.

## Funding

This work was supported by the 10.13039/501100001809National Natural Science Foundation of China (No. 82070705, 81770670, 81873595), Shanghai Municipal Key Clinical Specialty (China) (No. shslczdzk02503), Shanghai Science and Technology Talent Program (China) (No. 19YF1450300), Research Projects of Shanghai Science and Technology Committee of China (No. 17411972100), Shanghai Science and Technology Innovation Action Plan of Scientific Instruments and Chemical Reagents Project (China) (No. 24142201800), and 10.13039/501100004543China Scholarship Council (No. 202408310237).

## Conflict of interests

The authors have no competing interests to declare.
